# The Skin Diseases of Infancy and Early Life

**Published:** 1889-11-23

**Authors:** 


### THE PRACTITIONERS' BOOKSHELF.

### THE SKIN DISEASES OF INFANCY AND EARLY LIFE.

The author* remarks that the skin diseases of early life present certain features, which entitle them to be studied clinically by themselves. Some are congenital, others are most common in the young, and some are very rare in early life. Moreover, we often find the skin in infancy striking the key-note of many diatheses which may affect the individual in after years. The author does not adopt the old classification of Willan, Bateman, and other writers, and we think properly so, for there are many objections to classifying skin diseases according to the appearances they present, as a papule may be vesicular to-morrow and pustular the next day. Dr. Campbell divides skin affections into those initiated in utero; eruptive fevers; chronic bacterial non-febrile diseases; diseases characterised by capillary flux in the tissue of the derma;

* By C. M. Campbell, M.D., C.M., Edin. Baillière, Tindall, and Cox, London. 1889.

diseases initiated by lesions of the epidermis and its involutions; neuroses; parasites, animal and vegetable. We turn to two of the most common skin diseases of childhood, eczema and ringworm. Of eczema, Dr. Campbell says that it "runs riot in some infants." The onset of acute infantile eczema is usually rapid, and commonly manifests its appearance by a patch of rose-red papules, punctate, or shading off into a general erythematous blush. The sensations of tingling, heat, and itching are probably very severe, as evidenced by the poor sufferer's cries, and by the scratching provoked. The practitioner is frequently confronted by the fact that eczema may occur a few weeks after vaccination, the latter, of course, being held by the ignorant to be the cause. But we have only to turn to the large number of infants who are attacked with eczema before vaccination to see how utterly groundless the accusation is. The irritant mode of washing and dressing which is too much in vogue with British mothers and nurses are often exciting causes of eczema. So is the premature giving of animal food. The obvious indications of treatment are to remove all discoverable irritants from without or from within, to discourage hyperæmia, and to rest the affected parts. Dr. Campbell has seen good results from lotions containing boracic acid, boro-glyceride, tincture of opium, hydrocyanic acid, oxide of zinc, etc. But the use of lotions requires careful and constant supervision, and they should not be continuously applied more than a few days, for the first good effects often pass away. As useful dusting powders may be mentioned the preparation of Fuller's earth known as cimolite. Highly-scented perfumers' powders should never be used. But some cases do better under ointments. Equal parts of lanoline and benzoated lard form an useful basis, to which may be added active ingredients as ichthyocol, resorcin, boracic acid, oleate of zinc. If there is profuse discharge, the ointment should be made antiseptic by carbolic acid. As regards washing the au hor observes, that when there is slight discharge or none at all, the dressed surface should be left undisturbed as much as possible, but when the discharge is profuse and crusts gather quickly, it is necessary to get rid of the debris. This is best done by applying carbolised olive oil with a shaving-brush, and afterwards a poultice. As internal medicines, over-fed and plethoric infants should be given one or two grains of grey powder every night, and magnesia in the morning. In the diet no preparation of meat should be allowed. "In ill-fed cases cod-liver oil is invaluable."

With respect to tinea, or ringworm of the scalp, Dr. Campbell says, "If we see a case consisting of one isolated patch of recent growth and moderate size, it may suffice to begin by cutting the hair quite short for a clear inch round the margin." If there are several patches the head should be shaved, or if shaving is painful the hair should be cut as short as possible. The author believes the disease may be eradicated without depilation. After the head has been cropped or shaved it may be necessary to apply a poultice. The scalp should then be swabbed with liquor potassæ, and after ten minutes well shampooed with carbolic or terebene soap and hot water, using a soft nail-brush. A clear, dry surface being obtained, glycerine and corrosive sublimate may be used, and a close-fitting cap should be worn. The author does not mention goa powder or its preparations, which we believe to be the best application for ringworm of the scalp.

Chapter IX., and last in the book, is headed, "Formulæ and Notes upon Therapeutic Agents." Under the heading of each disease are given the prescriptions referred to in the text, and recommended by the author, while some remarks on clothing and diet close the book. It is not too much to say that Dr. Campbell's "Skin Diseases of Infancy and Early Life" is an excellent little book, and it should be in the possession of every practitioner liable to be consulted for such ailments.

